# Influence of hybrid assistive limb gait training on spatial muscle activation patterns in spinal muscular atrophy type III

**DOI:** 10.12688/f1000research.50951.2

**Published:** 2021-05-21

**Authors:** Yuichi Nishikawa, Kohei Watanabe, Naoya Orita, Noriaki Maeda, Hiroaki Kimura, Shinobu Tanaka, Allison Hyngstrom

**Affiliations:** 1 Faculty of Frontier Engineering, Institute of Science and Engineering, Kanazawa University, Kanazawa, Ishikawa, 920-1192, Japan; 2 Laboratory of Neuromuscular Biomechanics, School of Health and Sports Sciences, Chukyo University, Nagoya, Aichi, 470-0393, Japan; 3 Division of Rehabilitation, Department of Clinical Practice and Support, Hiroshima University Hospital, Hiroshima, Hiroshima, 734-8551, Japan; 4 Division of Sports Rehabilitation, Graduate School of Biomedical and Health Sciences, Hiroshima University, Hiroshima, Hiroshima, 734-8551, Japan; 5 Department of Rehabilitation Medicine, Hiroshima University Hospital, Hiroshima, Hiroshima, 734-8551, Japan; 6 Department of Physical Therapy, Marquette University, Milwaukee, Wisconsin, 53233, USA

**Keywords:** Gait training, Rehabilitation, Spinal muscular atrophy type III, Electromyography, Hybrid assistive limb

## Abstract

**Background: **Despite the potential benefits, the effects of Hybrid Assistive Limb (HAL) gait training on changes in neuromuscular activation that accompany functional gains in individuals with spinal muscular atrophy (SMA) type III is not well known. In this article, we quantify the effects of HAL gait training on spatial muscle activity patterns in a patient with SMA type III using multi-channel surface electromyography (SEMG).

**Methods: **A 21-years old male (168 cm, 47.8 kg) with spinal muscular atrophy type III, when diagnosed at 18-years old by genetic screening, participated in this case study. Although he presented with forearm distal muscle weakness, atrophy of the intrinsic muscles of the hand, and neuromuscular fatigue, his activities of daily living is independent. The patient underwent a separate, single 33-minute session of both HAL and treadmill gait training. To evaluate the coefficient of variation (CoV) of force and alterations in the SEMG spatial distribution patterns, modified entropy and CoV of root mean square (RMS) were calculated from the vastus lateralis (VL) muscle before and after the intervention of HAL and treadmill gait training. Each training session was separated by a period of one month to avoid cross-over effects.

**Results: **There was a greater decrease in the ΔCoV of force and an increase in the magnitude of whole VL muscle activation from pre-intervention to post-intervention with the HAL gait training as compared to the treadmill gait training. In response to only HAL gait training, the CoV of RMS was higher, and the modified entropy was lower post-intervention than pre-intervention.

**Conclusions:** Our results support the notion that HAL gait training has a positive benefit on motor output not only in the magnitude of SEMG generated but also the patterns of neural activation.

## Introduction

Several clinical trials of robot-assisted gait training have been reported in neurology patients. ^1– 4^ In particular, the Hybrid Assistive Limb (HAL) gait training has resulted in improved gait and balance performance. ^1, 3, 5^ The HAL exoskeleton is completely driven by the patient’s own muscle activation, which is detected by surface electrodes on key lower extremity muscle groups. Thus, this self-initiated robotic-assisted movement has the potential to induce a somatosensory feedback-loop that enhances neural plasticity and locomotor function. ^6^ Although many previous studies have reported functional gains due to the HAL intervention (e.g., gait and balance), ^1, 3, 5, 7^ there are no studies that report its effects on neuromuscular activation likely to accompany functional gains. Understanding how HAL training changes neuromuscular activation patterns, will help in the evaluation and use of HAL training across various populations.

Multi-channel surface electromyography (SEMG) is a recently developed techniques used to evaluate single motor unit firing behavior and whole muscle EMG patterns of activity by using multiple electrodes arranged in a two-dimensional plane. ^8– 10^ This technique provides data on the spatial distribution of SEMG within a muscle. Previous studies have demonstrated that the spatial distribution of SEMG is altered by contraction levels or fatigue during isometric contraction. ^11, 12^ This phenomenon has been explained by a spatial inhomogeneity in the location of different types of muscle fibers ^13^ and a clustering of muscle fibers innervated by one motor unit in a limited territory. ^14– 16^ Previous studies have demonstrated that alterations in the multi-channel SEMG spatial distribution pattern can be explained by the physiological phenomenon of motor unit recruitment and suggested that the multi-channel SEMG spatial distribution pattern could be used to study changes in motor unit recruitment patterns. ^15, 17^ In addition, previous studies have used metrices such as the inhomogeneity of the spatial distribution pattern of SEMG (EMG variables; modified entropy and coefficient of variation (CoV) of root mean square (RMS) ^11, 18^ to quantify neuromuscular fatigue and the influence of force generation. The assessment of motor unit recruitment is very important for determining the effect of neurorehabilitation on neuroplasticity in patients with several types of neurological conditions. Thus, we considered that this technique (i.e., multi-channel SEMG) could be employed as a new assessment tool to evaluate changes in muscle activation as a metric of neuroplasticity in response to HAL training.

Individuals with spinal muscular atrophy (SMA) type III present with progressive proximal weakness of the legs more than arms, and the leg weakness is so profound that it can necessitate the long term need of a wheelchair for mobility. ^19^ Rehabilitation is one treatment for SMA type III. The main objectives for rehabilitation of individuals with SMA type III who are still able to walk are to maintain, restore or promote function, mobility, and adequate joint range, and improve balance and endurance. ^20^ HAL gait training has been an insurance supported therapy since January 2016, and SMA, amyotrophic lateral sclerosis, spinal bulbar atrophy, Charcot-Marie-Tooth disease, inclusion body myositis, distal myopathy, and congenital myopathy are targeted disease populations. Although approved by insurance, there is limited information on how the effectiveness of HAL training compares to other forms of therapy. Specifically, there is no report which compares HAL and conventional treadmill gait training on neuromuscular activation in SMA patients that likely accompany improvements in walking function. Therefore, the aim of this case report to quantify the effects of a single session of HAL gait training on SEMG spatial distribution patterns in a person with spinal muscular atrophy type III. We hypothesized that HAL gait training would induce more heterogenous muscle activation reflecting more varied motor unit recruitment than treadmill gait training.

## Methods

### Participant background and examination

A 21-year-old male (168 cm, 47.8 kg) with spinal muscular atrophy type III when diagnosed at 18-years old by genetic screening participated in this study. Written informed consent was obtained from the patient to perform the two types of gait training and to publish the obtained his anonymized data to be published in this article. All procedures were performed in accordance with the Declaration of Helsinki and were approved by the Hiroshima University’s Committee on Ethics in Research (approval Number No. E-53). He presented with forearm distal muscle weakness, atrophy of the intrinsic muscles of the hand, and neuromuscular fatigue. The patient’s baseline physical performance data are shown in Table 1. The age-matched normative values for knee extension strength and six minutes walking test (6MWT) are 2.82±0.38 Nm/kg and 637 m. ^21, 22^ The participants’ knee extension strength (Right: 1.59 Nm/kg, Left; 1.32 Nm/kg) and 6MWT (567 m) were lower than the above value, and we have chosen the weaker leg to measure EMS recording.

**Table 1.** Participant’s physical function.

Variables	
Muscle strength Knee extension torque, Nm/kg	Rt: 1.59, Lt: 1.32
10m walk speed, sec	5’28, 15 steps
Timed up and Go test, sec	5’53
Single leg standing, sec	Rt: 60<, Lt: 20’46
6 minutes walking test, m	567

The participant performed maximal isometric voluntary contractions (MVC) of the knee extensors bilaterally at the pre- and post-intervention. Isometric knee extension was performed using a Biodex system (Biodex System 4; Biodex Medical Systems, Shirley, NY, USA). During contractions, both the hip and knee extension angles were positioned at 90 degrees. The MVC involved a gradual increase in knee extension torque from 0 Nm to his maximum torque over three seconds, with the maximum torque being held for two seconds. The participant performed at least two MVC trials with > 120 s of rest between trials and a warm-up for 10 min, including indoor walking and lower limb stretching before the MVC measurement. ^23^ The peak MVC torque was used as the maximal effort and to calculate the target torque for the isometric sub-maximal ramp-up contractions. After MVC measurements, the participant performed an isometric submaximal ramp-up contraction of the knee extensors in the weaker leg one time from 0% to 80% of the MVC force with an increase rate of approximately 10% of the MVC per sec. ^24, 25^ The participant-generated torque and target torque were displayed on a computer monitor. Prior to motor testing, the participant practiced the MVC and sub-maximal ramp-up contraction at least 10 min before the motor testing session began. The CoV of force (standard deviation (SD)/mean x 100, CV force) was calculated from the ramp up contraction task.

### EMG recording

During the sub-maximal contraction, multi-channel SEMG signals were detected from the weaker side of vastus lateralis (VL) muscle using a semi-disposable grid of 64 electrodes (ELSCH064NM2; OTBioelettronica, Torino, Italy) according to the same procedures used in previous studies. ^23, 24, 26, 27^ The grid consisted of 13 columns and five rows of electrodes (diameter, 1mm; inter-electrode distance, 8mm in each direction), with one missing electrode in the upper left corner. The participant’s thigh hair was removed, and the skin was cleaned with alcohol. The electrode was attached to the skin with a bi-adhesive sheet (KITAD064NM2; OTBioelettronica) after a conductive paste (Elefix Z-181BE; NIHON KOHDEN, Tokyo, Japan) was applied. The center of the electrode grid was positioned at the center of the line between the superior lateral edge of the patella and the greater trochanter protuberance. The columns of the electrode grid were placed parallel to the longitudinal axis of the VL muscle. A reference electrode was attached at the anterior superior iliac spine. ^23, 24, 26, 27^ All procedures were performed by the same investigator.

Monopolar multi-channel surface EMG signals (64 channels) were amplified by a factor of 1000, sampled at 2048 Hz, and digitized by a 12-bit analog-to-digital converter (EMG-USB2+, OTBioelettronica). The recorded monopolar signals were off-line bandpass filtered (10-500 Hz) and transferred to analysis software (MTALAB 2019a, MathWorks GK, MA, USA). Bipolar multi-channel SEMG signals (n = 59) along the columns were obtained from the 64 electrodes. The EMG signals were divided in epochs of one second centered at each 10% of MVC force increment from 10% to 80% of the MVC ramp-contraction to calculate the root mean square (RMS). ^24^ Since the selected ramp rate was 10% of the MVC force per second, one epoch of the sampled signal was overlapped by 0.5 seconds between neighboring torque levels. We normalized the RMS estimates to the values obtained pre-intervention for each torque level.

To characterize the heterogeneity in the multi-channel SEMG spatial distribution pattern at each epoch, we determined the modified entropy and correlation coefficients. The modified entropy of the spatial distribution of the ΔSEMG amplitude was calculated over a 1-s epoch taken at 10% to 80% of the MVC during the ramp-up contraction. According to methods published by Farina et al., modified entropy was defined as the entropy of the signal power as follows: ^11^

$$E=-\sum_{i=1}^{59}p{\left(i\right)}^{2}\log_{2}p{\left(i\right)}^{2},$$

where *p*( *i*) is the square of the RMS value of channel *i* divided by the sum of the squares of all 59 RMS values at the given contraction level. Therefore, *p*( *i*) ^2^ represents the normalized power of each channel. Modified entropy is the normalized power of the EMG signal across the array and reflects the heterogeneity in the muscle activity. The CoV of the RMS was defined as the quotient of the SD of the Δ59 RMS measurements and the average of Δ59 RMS measurements at each given torque level. ^24^ Both a decrease in modified entropy and an increase in CoV of RMS indicate increased heterogeneity in the multi-channel SEMG spatial distribution pattern within the electrode grid. ^12, 18^ Measures of the CoV of the RMS and modified entropy provide insight into how the nervous system regulates muscle activation across the muscle irrespective of the magnitude. ^28^

### Intervention

The patient performed two types of gait training separated by a period of one month between training to avoid training effect.

***HAL gait training***

The patient performed gait training using the HAL for 33 minutes (gait distance about 2200 m). The double leg type HAL was used for gait training, which was performed with two physical therapists for the operation of the HAL commands and support of the patient. During HAL gait training, a walking hoist (ALL IN ONE walking hoist, ROPOX, Denmark) was used to prevent falls and adjust the patient’s posture.

***Treadmill gait training***

The patient performed gait training using the treadmill for 33 minutes (gait speed 4.0 km/h, gait distance about 2200 m). During gait training, Alter-G (Alter-G, Inc., Fremont, CA, USA) was used to prevent falls and adjust the patient’s posture without providing weight bearing and clinician’s support to leg movements.

## Results

**Figure 1.**
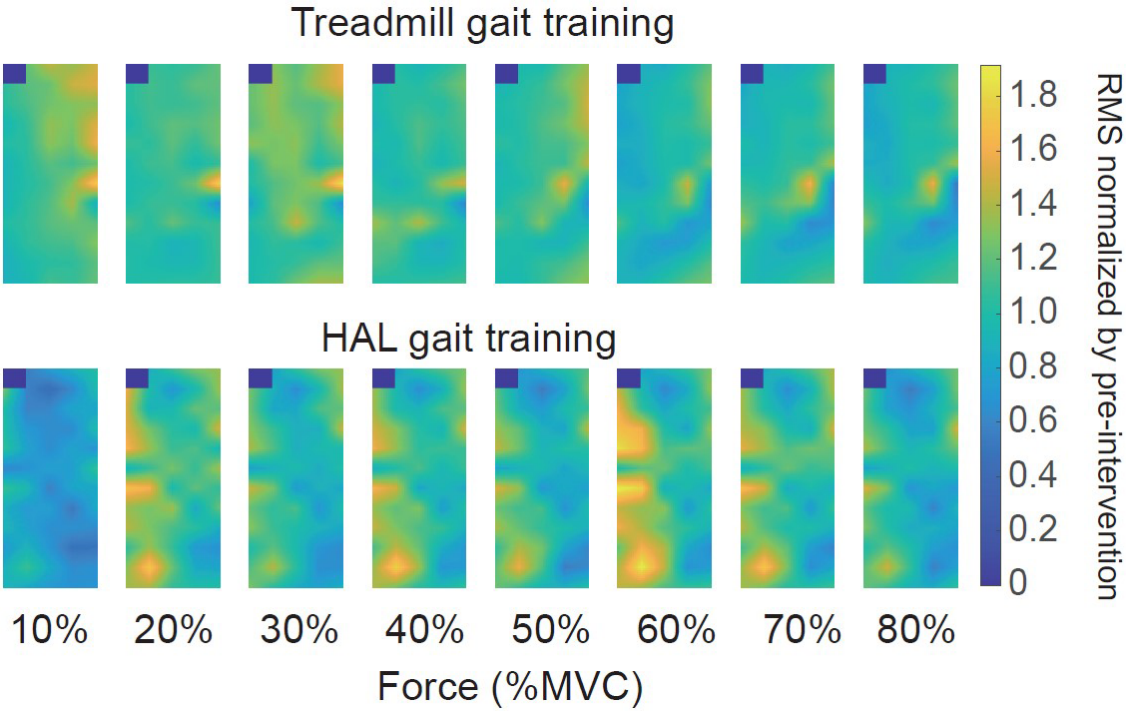
The representative color map during isometric knee extension task. (upper panel) treadmill gait training, (lower panel) HAL gait training. Differences in the multi-channel surface electromyography spatial distribution patterns at each torque level were observed between the treadmill and HAL gait training in these representative data.

**Figure 2.**
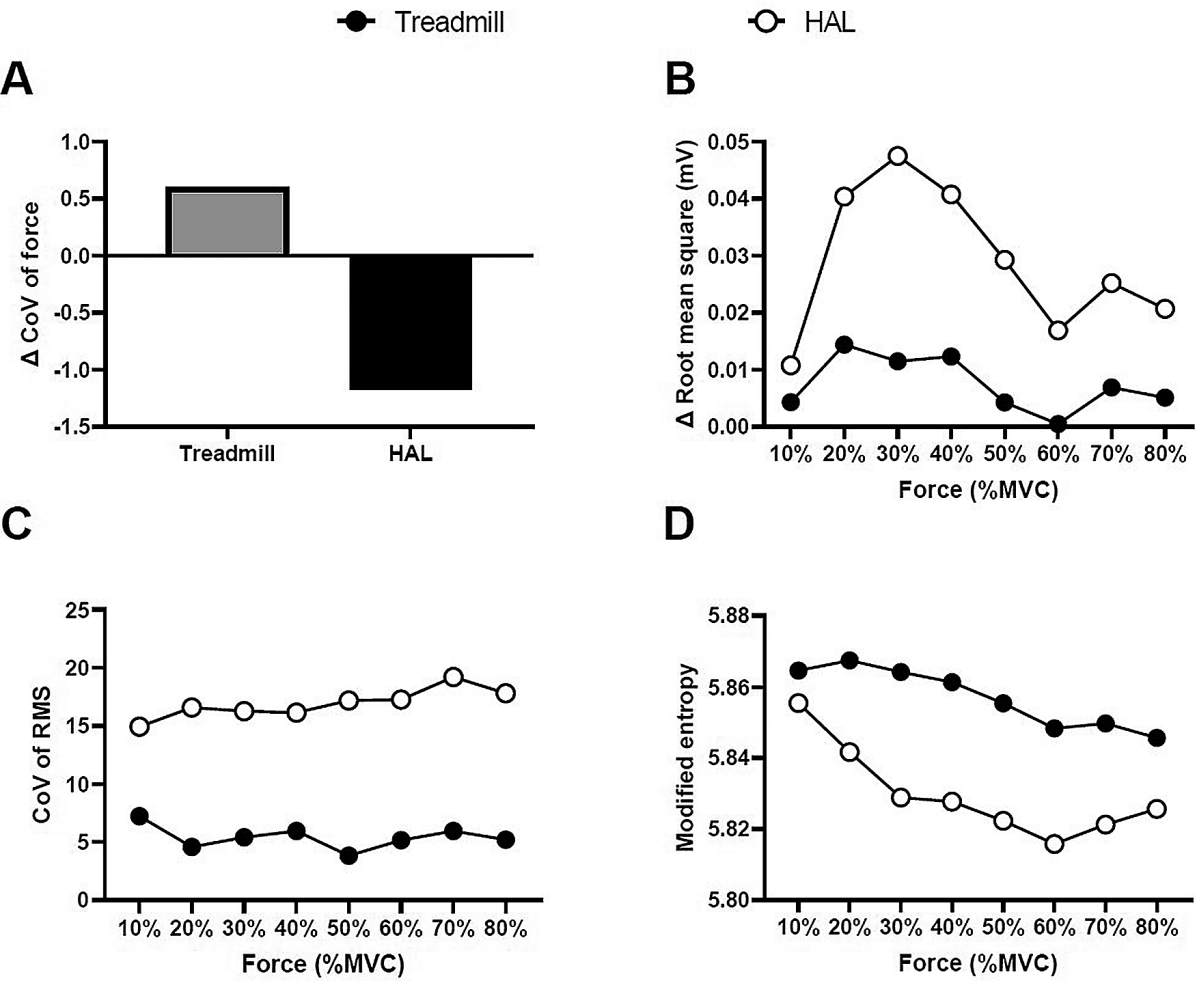
The force and electromyography variables. The Δ coefficient of variation (CoV) of force (A), Δ root mean square (B), CoV of RMS (C), and modified entropy (D) of the treadmill gait training and HAL gait training.

As a neuromuscular activation assessment, multi-channel SEMG was performed on the patient before and after both gait training. Figure 1 illustrates the representative multi-channel SEMG amplitude color maps for treadmill and HAL gait training. We normalized the CoV of force and RMS estimates to the values obtained pre-intervention for each torque level. Differences were observed in the multi-channel SEMG spatial distribution pattern at each contraction torque between HAL and treadmill gait training. There was a greater decrease ΔCoV force with the HAL gait training than for treadmill gait training ( Figure 2A). The increase in the magnitude of muscle activation from pre-intervention to post-intervention was greater for HAL gait training than for treadmill gait training ( Figure 2B). The CoV of the RMS and modified entropy at each torque in both gait trainings are shown in Figure 3. For HAL training, both the CoV of the RMS were higher and the modified entropy was lower as compared to treadmill gait training ( Figure 2C and D). No adverse events were observed with either intervention. He did not experience worsening of the symptoms during each gait training.

## Discussion

We applied multichannel SEMG as a neuromuscular activation assessment of HAL training. In addition to the overall increase in EMG activity, the HAL gait training resulted in decrease force fluctuation, and greater heterogeneity in spatial muscle distribution pattern from pre-intervention to post-intervention than for the treadmill gait training.

We used two parameters to quantify the multi-channel SEMG spatial distribution pattern: modified entropy and the CoV of the RMS. A decrease in the modified entropy and an increase in the CoV of the RMS are consistent with increased heterogeneity in the multi-channel SEMG spatial distribution pattern within the electrode grid. ^25^ The SEMG spatial distribution pattern can be altered by contraction levels or fatigue during isometric contraction. ^11, 12^ Measures of the CoV of the RMS and modified entropy provide insight into how the nervous system regulates muscle activation across the muscle irrespective of the magnitude. ^28^ This phenomenon has been explained by spatial heterogeneity in the location of different types of muscle fibers ^13^ and a clustering of muscle fibers innervated by one motor unit in a limited territory, ^14, 15^ and a previous study reported that alterations in the multi-channel SEMG spatial distribution pattern can be explained by the physiological phenomenon of motor unit recruitment patterns. ^17^ The results of the present report for the HAL training intervention showed that the CoV of the RMS was higher, and the modified entropy was lower than for treadmill gait training. These findings suggest that spatial motor unit activity patterns were enhanced by HAL training. Changes in the heterogeneity of SEMG were accompanied by a greater decreased in the Δ CoV force for the HAL gait training as compared to the treadmill gait training. This finding suggests that the participant was able to perform the submaximal ramp up task smoothly by altered spatial motor unit activity patterns.

The HAL gait training offers the possibility to monitor muscle contractions via SEMG at the extensor and flexor muscle region of the lower extremities. ^5, 29^ The voluntary drive and normalized motion assistance provided by the external device likely form the foundation for a proprioceptive feedback loop for patients with lesions involving the sensory pathway and not facilitated as well with treadmill training. The neural activity and repeated execution of specific tasks promote learning and lead to the reinstatement or restructuring of appropriate proprioceptive feedback. ^30, 31^ Here, we are the first to show that HAL gait training induced an alteration muscle activation patterns due to a single session of HAL training. Moreover, based on our findings, HAL gait training may be more effective in evoking changes in neuromuscular activation than conventional gait training. Future studies will examine the relationship between changes in gait and muscle activation using the HAL intervention across multiple sessions.

This report has several limitations. First, report included only one patient with spinal muscular atrophy type III. Future large-sample studies and studies of patients with various neurological conditions (e.g., amyotrophic lateral sclerosis, Parkinson’s disease, and stroke) are needed to clearly understand how generalizable the effects of HAL gait training on neuroplasticity. Second, this report only included data following a single session. Future studies will examine how factors such as dose, frequency, and intensity of HAL training affect changes in function and neuromuscular activation.

## Conclusions

The present case report showed that HAL gait training was more effective in changing metrics of neuromuscular activation of the knee extensors than treadmill gait training despite similar training durations. These data suggest that the modes of gait training which incorporate both voluntary activation and guided movement of the legs may facilitate recovery of function.

## Consent

Written informed consent for publication of their clinical details and/or clinical images was obtained from the patient/parent/guardian/relative of the patient.

## Data availability

### Underlying data

Figshare: EMG data of VL muscle, https://doi.org/10.6084/m9.figshare.14113178.v1. ^32^

Data are available under the terms of the Creative Commons Attribution 4.0 International license (CC-BY 4.0).
