# Supplementary material for: Influence of hybrid assistive limb gait training on spatial muscle activation patterns in spinal muscular atrophy type III
Source: F1000Res. 2021 May 21;10:214. Originally published 2021 Mar 16. [Version 2] doi: 10.12688/f1000research.50951.2 (PMC8258705; doi:10.12688/f1000research.50951.2)
Supplement: Peer review history

# Peer review: Reviewer response for version 2

**DOI:** 10.5256/f1000research.56538.r88730

**Published:** 2021-07-05

**Authors:** Corrado Cescon

**Affiliations:** 1 Rehabilitation Research Laboratory 2rLab, Department of Business Economics, Health and Social Care, University of Applied Sciences and Arts of Southern Switzerland, Manno, Switzerland

The manuscript describes the multichannel EMG analysis of the limb muscle of a patient with muscle atrophy. The patient performed ramp contractions and EMG maps were analyzed computing entropy. The methods are described accurately and the technology used is solid as well as the equipment used. The results are interesting, well commented and well described. The present reviewer has no additional request for changes.

Is the work clearly and accurately presented and does it cite the current literature?

Yes

If applicable, is the statistical analysis and its interpretation appropriate?

Yes

Are all the source data underlying the results available to ensure full reproducibility?

Yes

Is the study design appropriate and is the work technically sound?

Yes

Are the conclusions drawn adequately supported by the results?

Yes

Are sufficient details of methods and analysis provided to allow replication by others?

Yes

Reviewer Expertise:

Electromyography, biomechanics, rehabilitation

I confirm that I have read this submission and believe that I have an appropriate level of expertise to confirm that it is of an acceptable scientific standard.

# Peer review: Reviewer response for version 1

**DOI:** 10.5256/f1000research.54046.r83012

**Published:** 2021-04-27

**Authors:** Redha Taiar

**Affiliations:** 1 Physical and Rehabilitation Medicine Department, Sebastopol Hospital, University of Reims Champagne-Ardenne, Reims, France

The objective of this study was to quantify the effects of HAL gait training on spatial muscle activity patterns in a patient with SMA type III using multi-channel surface electromyography (SEMG). The authors support in conclusion the notion that HAL gait training has a positive benefit on motor output not only in the magnitude of SEMG generated but also the patterns of neural activation.

 The approach of the study appears very original and the contents of the manuscript are quite interesting by his methodology and through the tools of quantification used.

 The manuscript reads smoothly and is easy to understand. The aims, scope, and results of the study are clearly stated. I have very much enjoyed reading this paper. I find it interesting and clearly written, and satisfying also all the other publication criteria of the “F1000Research”. The study provides a very valuable addition to this line of research, and adds relevantly to the subject with additional original findings. I thus find that this paper definitively delivers results that will surely be of interest to the readership of the “F1000Research”. I recommend the publication of this interesting paper after more details concerning the Ethical Committee “ *All procedures were performed in accordance with the Declaration of Helsinki and were approved by the Hiroshima University’s Committee on Ethics in Research (approval Number No. E-53). Our institution does not require ethical approval for reporting individual cases or case series*”. The details are not clear and must be re writting. For instance " *Our institution does not require ethical approval for reporting individual cases or case series*” what I recommend to the authors to delete.

Is the work clearly and accurately presented and does it cite the current literature?

Yes

If applicable, is the statistical analysis and its interpretation appropriate?

Yes

Are all the source data underlying the results available to ensure full reproducibility?

Yes

Is the study design appropriate and is the work technically sound?

Yes

Are the conclusions drawn adequately supported by the results?

Yes

Are sufficient details of methods and analysis provided to allow replication by others?

Yes

Reviewer Expertise:

biomechanics, health disease and rehabilitation

I confirm that I have read this submission and believe that I have an appropriate level of expertise to confirm that it is of an acceptable scientific standard.

# Response

**Published:** 2021-04-28

**Authors:** Yuichi Nishikawa

**Affiliations:** Kanazawa University, Japan

Dear Prof. Redha Taiar,

 Thank you for reviewing this paper and helpful comments.

 We have removed the bellow sentence.

 " *Our institution does not require ethical approval for reporting individual cases or case series*”
